# Consolidation process of uncemented backfill slurry in a mine stope considering hydro-geotechnical properties of rockmass in adjacent stopes

**DOI:** 10.1038/s41598-025-08369-5

**Published:** 2025-07-06

**Authors:** Guangsheng Liu, Qinghai Ma, Xiaocong Yang, Lijie Guo, Li Li

**Affiliations:** 1 BGRIMM Technology Group, Building 23, Zone 18 of ABP, No. 188, South 4Th Ring Road West, Beijing, 100160 China; 2 National Center for International Research On Green Metal Mining, No. 22, Beixing Road East, Daxing District, Beijing, 102628 China; 3 Research Institute On Mines and Environment (RIME UQAT-Polytechnique), Department of Civil, Geological and Mining Engineering, École Polytechnique de Montréal, Montréal, Québec H3C 3A7 Canada

**Keywords:** Backfill slurry, Consolidation, Pore water pressure, Effective stress, Rockmass, FLAC3D, Engineering, Mathematics and computing

## Abstract

In open stoping with subsequent backfill mining, the filled slurry in one stope is typically surrounded by the rockmass in adjacent stopes. The rockmass generally contain geological faults and joints that can serve as seepage pathways for pore water within the backfill slurry during consolidation process. However, these impacts from the adjacent rockmass were usually simplified to an impermeable or permeable boundary in previous studies. In this paper, numerical modeling with FLAC3D was conducted to investigate the influences of hydro-geotechnical properties of surrounding rockmass on consolidation process of uncemented backfill slurry in a vertical stope. Results show that the pore water pressure (PWP) and effective stresses of backfill slurry confined by rockmass are consistently higher than those obtained by assuming fully permeable boundaries but lower than those derived from impermeable boundary assumptions. A five-fold difference in peak PWP and effective stresses occurs when the rockmass hydraulic conductivity varies from 10^–8^ m/s to 10^–5^ m/s. It is reasonable to simplify the rockmass with a low hydraulic conductivity (≤ 10^–8^ m/s) as an impermeable boundary and that with high hydraulic conductivity (≥ 10^–5^ m/s) as a permeable boundary. Additionally, a higher porosity and lower initial saturation of the adjacent rockmass promote both PWP dissipation and effective stresses development in backfill slurry, but their influences are less pronounced compared to the effect of hydraulic conductivity. Furthermore, the study discusses the influences of the hydro-geotechnical properties of adjacent rockmass on the lateral earth pressure coefficient of consolidated backfill which is an important parameter for analytical models of stress distribution within backfill. The findings are expected to provide valuable insights into the consolidation behavior of backfill slurry under field conditions and contribute reliable method for barricade design.

## Introduction

Valuable minerals are extracted mostly from underground stopes, which generates large voids and produces substantial amounts of solid waste, including tailings and waste rocks. To address these issues, mining with backfill has been increasingly adopted in mines worldwide, which allows the tailings to be backfilled into the excavated stopes, thereby reducing the surface disposal of mine wastes, improving ground stability around underground voids and increasing ores recovery rate^1–3^.

Mine tailings blended with water and/or binders are transported from surface backfill plants primarily by pipelines and then poured into underground stope voids. Prior to filling, the accesses to the stopes must be blocked using man-made structures known as barricades, which are designed to retain the backfill tailings slurry. During and after filling process, the barricades must remain stable until the backfill slurry have reached the desirable consolidation state under self-weight loading. Despite this clear purpose, accidents of barricades cracking occur occasionally, often caused by the pressure exerted by the unconsolidated slurry. Such incidents not only pose significant safety hazard to miners and equipment but also lead to considerable economic losses to the mine in cleaning up the rushed-out slurry during operational shutdowns^4^. Therefore, it is significant to correctly evaluate the pore water pressure (PWP) dissipation of the backfill slurry especially in large scale stopes of mass mining. Prolonged high PWP levels can exacerbate the risk of serious barricade failures in underground workplaces, further emphasizing the importance of investigating the consolidation behavior of backfill slurry^4^.

To analytically evaluate the PWP during the consolidation process of backfill slurry in mine stopes, one can refer to the Gibson model^5^. This model was initially proposed to investigate the self-weight consolidation of an accreting clayey layer based on one dimensional consolidation assumptions of Terzaghi’s theory^6^. The quasi-analytical solution of the Gibson model contains an integral part that can only be numerically evaluated to calculate the PWP of ongoing slurry sedimentation. This limitation was addressed by Zheng et al.^7,8^, who formulated the true analytical equations for the consolidation of slurried materials with pervious or impervious base. In addition, Fahey et al.^9^ updated the Gibson model by modifying the constant coefficient of consolidation to a variable value. This adjustment was based on experimentally tested permeability and constrained modulus data, which varied with consolidating time of slurried materials. Cui and Fall^10^ proposed coupled analytical models to calculate the PWP and effective stress of paste backfill, incorporating consolidation behavior under thermal, hydraulic, mechanical and chemical (THMC) processes. While the Gibson model and its following modifications provide a reasonable representation of the one-dimensional consolidation process for backfill slurry, they typically assume simplified stope conditions. Specifically, these models consider the stope to have impermeable side walls, only allowing water to drain freely from the top and/or bottom of the backfilled stope.

Numerical modeling has proven to be an effective and efficient tool to investigate the consolidation process of backfill slurry in mine stopes. Helinski et al.^11,12^ utilized the Minefill-2D code to study the coupled behavior of consolidation and hydration in cemented tailings backfill. In their models, the drainage was allowed through the barricade, while side walls of the modeled stope were set as impermeable. El Mkadmi et al.^13^ investigated the consolidation behavior of backfill slurry using SIGMA/W. Their models applied free drainage conditions at the stope floor and the top surface of the slurry, while the side walls were treated as waterproof boundaries. Li^14^ also employed SIGMA/W to numerically model the effects of wick drains on the drainage process of backfill slurry within stopes. The wick drains together with the top and base planes of the stopes were simulated with free drainage boundaries, while the side walls of stopes were considered impervious. Doherty^15^ conducted a numerical study using ABAQUS to evaluate the influence of soil water retention properties on PWP and effective stress within a single mine stope. The model incorporated free drainage conditions at the top surface of the backfill and the barricade, while the stope base and side walls were modeled as impervious. Veenstra^16^ developed a numerical model using FLAC3D to examine the pressure exerted by early-age paste backfill on the barricade during stope filling, with the side walls of the modeled stope also treated as impermeable. As can be seen from these previous studies, numerical simulations of the consolidation behavior of backfill slurry mainly focused on individual mine stopes. The influence of rockmass in adjacent stope has been arbitrarily simplified as either impermeable or permeable drainage conditions along the side walls. The consolidation process of backfill slurry in one stope surrounded by rockmass in adjacent stope has never been modeled and simulated in previous studies.

A large number of physical model tests are also reported to study the consolidation behavior of backfill slurry by setting reasonable conditions based on field backfill properties in mines^17–20^. These tests commonly employ columns to simulate individual mine stopes, with the side walls of the columns typically treated as impermeable. This setup implies that the modeled stope represents a scenario where pore water within the backfill slurry cannot drain through the side walls into adjacent stopes. Additionally, field monitoring of PWP and total stress of backfill slurry has been conducted at many mines^21–24^. However, due to the inherent limitations and lack of operational flexibility in field monitoring, the results generally only reflect the consolidation process of backfill slurry within an individual stope.

Previous studies have significantly advanced the understanding of the consolidation process of backfill slurry in isolated mine stopes. Nevertheless, the influence of adjacent stopes on the consolidation process has yet to be addressed. In practice, given the multiple steps of excavating and filling procedures of the divided mine stopes in open stoping with subsequent backfill mining methods, the backfill slurry in one stope is typically confined by the rockmass and/or cured backfill body in adjacent stopes. The rockmass usually contain geological faults, joints and/or mining induced cracks that can serve as seepage pathways for pore water within the backfill slurry^25^. Clearly, the hydro-geotechnical properties of adjacent rockmass in nearby stopes, such as permeability and porosity, can significantly affect PWP dissipation and consolidation process of backfill slurry. Despite this, the rockmass in adjacent stopes has typically been modeled as either an impermeable or fully permeable boundary for backfill slurry. The influence of the hydro-geotechnical properties of the surrounding rockmass on the stress state and consolidation behavior of backfill slurry remains unstudied and unevaluated.

In this study, numerical simulations were first conducted with FLAC3D to investigate the consolidation behavior of uncemented backfill slurry in a vertical mine stope considering the hydro-geotechnical properties of rockmass in adjacent stopes, including permeability, initial saturation, and porosity. The influence of these hydro-geotechnical properties on PWP and total and effective stresses within the backfill slurry were comprehensively analyzed. Additionally, consolidation processes of the backfill slurry in stopes with impermeable and permeable side walls were simulated for comparison. Numerical outcomes on PWP are validated against the analytical results from the Gibson model^5^ and the in-situ monitoring results reported by Helinski et al^23^. Some simulated effective stresses are further compared with the analytical solution proposed by Li and Aubertin^26,27^. The findings are expected to provide valuable insights into the consolidation behavior of backfill slurry under field conditions and contribute to more reliable method for barricade design. The influences of cement hydration on the consolidation of backfill slurry are not emphasized here but with necessary discussions. Coupled analyses about the thermal, chemical or mechanical factors, and the man-made drainage structures (e.g., wick drains, drainage holes, barricades arrangements) are not the concern of this paper.

## Numerical models and simulation schemes

### Model construction and boundary conditions

**Fig. 1:**
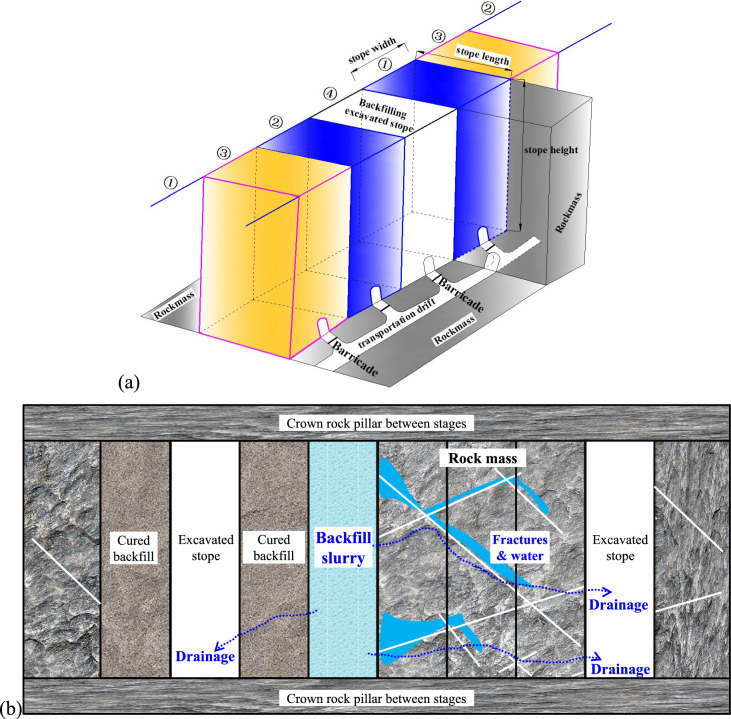
(**a**) A typical excavating and filling procedure of open stoping with subsequent backfill mining; (**b**) possible drainage paths for backfill slurry in one stope through adjacent rockmass and/or backfill.

As shown in Fig. 1a, in multiple excavating and filling steps of an open stoping with subsequent backfill mining method, the ore body is usually divided vertically into several stages, which are separated from each other by crown rock pillars to isolate the mining activities. At each stage, the ore rock will be further divided into primary stopes (stope ① and stope ②) and secondary stopes (stope ③ and stope ④) which can be synchronously excavated at interval of one stope or three stopes. For some multilayer or vein style ore-bodies, if the planned stopes are prospected to be barren rocks, these stopes will be left for ground control with no excavation, which will also have possible effects on the seepage paths of the tailings slurry in adjacent filled stopes.

The above excavating and filling procedures can lead the drainage boundaries and the consolidation processes of the backfill slurry in one typical vertical stope to the following conditions, especially after considering the lateral drainage boundaries of adjacent rock walls, as shown in (Fig. 1b):

If the backfill slurry during drainage and consolidation in one of a stope is confined by intact non-fractured rockmass or dense cemented fine tailings backfill, the drainage boundaries of the filled slurry along side walls will be nearly impermeable, which will result in a relatively slower consolidation and a higher PWP in the backfill slurry, because the bleed water from tailings slurry may only be able to drain through the barricades in the bottom of the stopes, or just accumulate on the top surface of tailing slurry to form a water pond^18,28^.

If the backfill slurry in a stope is surrounded by fractured rockmass or backfill bodies cured with coarse waste rocks, water from the tailings slurry will be able to flow across one or more stopes via possible drainage paths. In these cases, the hydro-geotechnical properties of the rockmass and/or existing backfill body are expected to have different effects on the consolidation process of the filled slurry, which are important and necessary to be investigated. However, these practical factors have rarely been reported in most previous numerical simulations^7,8,11–13,15,29–31^.

Except for the drainage conditions for side boundaries, the top surface of the filling slurry is fixed at zero PWP as usual, while the base plane of the stope is set to be impervious due to the crown rock pillars between stages which are commonly used for the top and base drainage boundaries in previous models^5,8,12,15,32^.

**Fig. 2:**
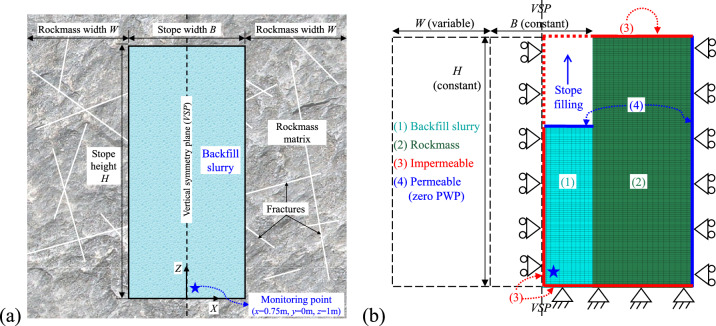
A gradually filled vertical stope considering adjacent rockmass: (**a**) idealized physical model; (**b**) numerical model constructed with FLAC3D.

As shown in Fig. 2a, a physical model consisting of backfill slurry and the adjacent rockmass was idealized based on the typical mining procedure of (Fig. 1). The excavated stope void gradually filled with slurry has a width *B*, a height *H* and confined by side rockmass with a width* W*. Accordingly, a plane strain numerical model was constructed in FLAC3D, as shown in (Fig. 2b), with a vertical symmetry plane (*VSP*) along the vertical central line of the stope width, to simulate the consolidating properties of the uncemented backfill slurry progressively deposited in the excavated stope void.

As shown in Fig. 2b, the mechanical and hydraulic boundaries of the numerical models were set as follows:

For the bottom plane of the backfill slurry and the adjacent rockmass illustrated by the red line, the mechanical boundary was set by fixing displacements in all directions together with impervious hydraulic simulations.

For the left side plane of filling slurry along *VSP* illustrated by the red line, only the horizontal displacement in its normal direction was fixed, and the hydraulic boundary was kept impermeable to simulate the symmetric issue.

For the right-side plane of rockmass matrix illustrated by the blue line, only the horizontal displacement in its normal direction was fixed, and the hydraulic boundary was set to be fully pervious (kept constant to zero PWP) to simulate the free drainage conditions of nearby excavated stope voids shown in (Fig. 1). This configuration is based on the commonly used primary-and-secondary mining sequence which allows the two stopes to be mined concurrently.

For the top surface of the rockmass matrix illustrated by the red line, which is free to move in all directions, the hydraulic boundary was set to be waterproof due to the crown rock pillar between the two mining stages shown in (Fig. 1b).

For the continuously deposited backfill slurry in the excavated stope void, the top surface of each newly added backfill layer (0.5m per layer in height) illustrated by the blue line was set to be fully pervious (zero PWP) and was allowed free deformations without fixed displacements. Besides, it should be noticed that after adding a new slurry layer on the previously deposited backfill layer, the zero PWP boundary on the top surface of the previous layer should be removed and re-assigned to the top surface of the newly added backfill layer.

Under the above mechanical and hydraulic boundaries, a series of sensitivity analysis about model meshing in FLAC3D were performed to ensure stable numerical results. The hexahedral elements were applied with 0.25 m in *Z*-direction and 0.5 m in *X*-direction per grid. A 0.25 m element in *Y*-direction (perpendicular to the page) was used for all models and set to a fixed *Y*-displacement and impervious boundary to simulate the plane strain problem.

### Material parameters and numerical cases

In all the numerical models here, the backfill slurry and rockmass were assumed to be homogeneous, isotropic elasto-plastic materials, obeying the Mohr–Coulomb criterion, with mechanical and hydraulic parameters as shown in (Table 1). These typical values were selected mainly based on the reports given in the previous literature^21,32–38^ and partly based on the authors’ experiences gained during the realization of several mining projects with backfill.

**Table 1:** Mechanical and hydraulic parameters of the backfill slurry and rockmass.

No	Items	Parameters	Symbols	Values	Unit	References
1	Uncemented backfill slurry	Dry density	*ρ*_df_	1500	kg/m^3^	^35^
2		Bulk modulus	*K*_f_	480	MPa	
3		Shear modulus	*G*_f_	360	MPa	
4		Cohesion	*c*_f_	0	kPa	
5		Internal friction angle	*ϕ*_f_	30	°	
6		Dilation angle	*ψ*_f_	0	°	
7		Hydraulic conductivity	*k*_f_	5 × 10^–6^	m/s	^33^
8		Porosity ratio	*n*_f_	50%	–	^34^
9		Initial saturation ratio	*s*_f_	100%	–	
10	Rockmass	Dry density	*ρ*_d_	2700	kg/m^3^	^35^
11		Bulk modulus	*K*	2.8	GPa	
12		Shear modulus	*G*	1.68	GPa	
13		Cohesion	*c*	9.4	MPa	
14		Internal friction angle	*ϕ*	38	°	
15		Dilation angle	*ψ*	0	°	
16		Hydraulic conductivity	*k*	***VAR***	m/s	^25^
17		Porosity ratio	*n*	***VAR***	–	
18		Initial saturation ratio	*s*	***VAR***	–	
19	Water	Density	*ρ*_w_	1000	kg/m^3^	Constant
20		Bulk modulus	*K*_w_	2 × 10^6^	kPa	
21	Other	Gravitational acceleration	*g*	10	m/s^2^	

The fluid flow through fractured rockmass can be numerically investigated with a discrete approach by modeling the drainage through each fracture in the rockmass, or with a continuum approach by averaging fracture parameters to quantify an equivalent homogeneous porous medium^39^. Generally, a continuum approach offers higher computational efficiency. In this study, since the numerical simulations focus on the consolidation process of the backfill slurry, the actual structural planes in real rockmass including geological joints and/or mining-induced fractures (as shown in Fig. 1) were not modeled in details. Instead, the rockmass was idealized as a homogeneous medium in FLAC3D characterized by the equivalent permeability, porosity, and initial saturation. This simplification was also adopted in previous numerical studies^25,39,40^.

The hydro-geotechnical properties of the rockmass were key variable factors schemed in numerical program of Table 2 which were considered to investigate their effects on the consolidation process of confined backfill slurry in a typical mine stope. Moreover, the stope width (*B* = 12m) and the final backfilled stope height (*H* = 30m) were held constant in all the numerical models of Case1, Case2 and Case3 shown in (Table 2). But the width of the rockmass,* W*, was varied in Case4 based on the proposed simulation program to represent the different seepage paths for backfill slurry drainage.

**Table 2:** Simulation scenarios for the consolidation process of backfill slurry in mine stopes considering the hydro-geotechnical properties of adjacent rockmass.

Cases		Properties of rockmass laterally confining backfill slurry				Hydraulic boundaries on side walls of filled stope
		Width *W* (m)	Hydraulic conductivity *k* (m/s)	Porosity ratio *n*	Initial saturation ratio *s*	
Case0	4, 5, 6	0	/	/	/	impermeable
Case1	7, 8	12	1 × 10^–5^, 1 × 10^–6^, 1 × 10^–7^, 1 × 10^–8^	25%	40%	Based on assigned hydraulic properties of the rockmass
Case2	10, 11	12	1 × 10^–6^	10%, 25%, 40%	40%	
Case3	12, 13	12	1 × 10^–6^	25%	40%, 60%, 80%, 100%	
Case4	15	12, 36	1 × 10^–6^	25%	40%	

## Validation on numerical simulated consolidation states of backfill slurry

### Simulated PWP compared with analytical results of the Gibson model

A world-recognized model to analytically calculate the PWP of accreting sediment layers consolidating with its increasing thickness was proposed by Gibson in 1958, which can be employed to investigate the consolidation process of the backfill slurry in mine stopes.

**Fig. 3:**
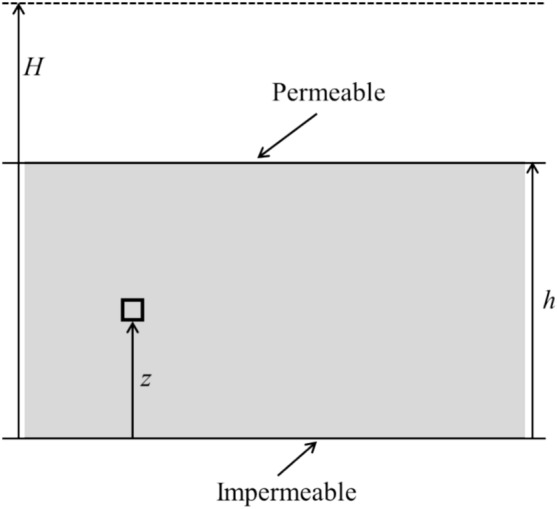
Gibson model for the self-weight consolidation of accreted sediment.

In analogy with the Gibson model^5^, Fig. 3 can be used to illustrate the deposition of backfilling slurry on an impermeable base of a mine stope. The initial thickness of the slurry at the start is zero. After a deposition time *t* (hour) and a constant increasing rate *m* (meter per hour, simplified as m/h), the backfilled slurry reaches a certain thickness *h* (m). Thus, the current height of the slurry can be expressed as *h* = *m* × *t*. Under the same increasing rate *m* in the vertical direction, the slurry will continue to rise until it reaches a final height of *H* (m).

As the height of the backfill slurry increases, the pore water can only drain from the top permeable surface in Gibson model with the assumed impermeable side and base boundaries. In this condition, Eq. (1) was proposed to calculate the excess PWP at an elevation *z* (m) and deposition time *t* (hour) in the backfill slurry.1

where *u* (kPa) is the excess PWP; *c*_v_ (m^2^/h) is the consolidation coefficient of the slurry; *γ′* = *γ*_f_ − *γ*_w_, *γ*_f_ and *γ*_w_ (kN/m^3^) are the unit weight of the filled slurry and the water, respectively.

Considering the zero PWP of the constant free drainage boundary at the top permeable surface of backfilled slurry, Eq. 1 can be solved to calculate the excess PWP *u*2

where *ξ* (dimensionless) is an integral variable (0 < *ξ* < ∞).

Gibson defined a dimensionless time factor *T* to further interpret the calculated results about the excess PWP at any accreted thickness *h* and relevant time *t* of the deposited slurry, which can be expressed as the Eq. (3).3

Before numerically investigating the consolidation process of backfill slurry in a mine stope considering the hydro-geotechnical properties of adjacent rockmass, the applicability and reliability of the FLAC3D program should be first validated. The Gibson model mentioned above can give analytical results of the PWP to verify the numerical outcomes.

The self-weight consolidation problem presented by Gibson was firstly modeled in FLAC3D. The width of the surrounding rockmass *W* was set to zero shown as the Case0 in (Table 2). The hydraulic boundaries include a fixed zero PWP (free drainage) at the top surface of each filled layer and impermeable side and base surfaces. Besides, the mechanical boundaries of the backfill slurry were set to be completely fixed at the bottom surface in all directions and fixed at the side walls in horizontal directions. These configurations were all consistent with the settings of Gibson’s analytical model.

The parameters used in the analytical and numerical models were shown in (Table 1). In FLAC3D models, the unit weight *γ*_f_ (kN/m^3^) and consolidation coefficient *c*_v_ of the simulated backfill slurry can be obtained by Eqs. (4) and (5).45

**Fig. 4:**
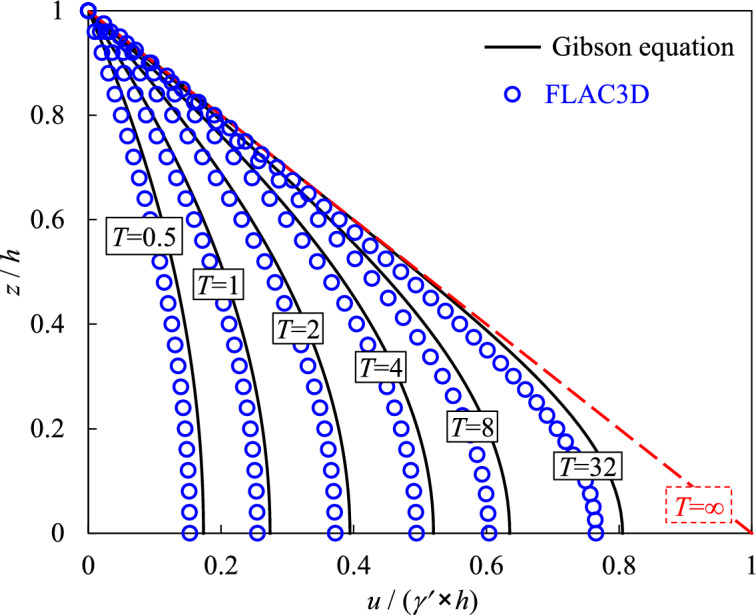
Comparisons of simulated PWP with the analytical results at different time factor *T*.

Figure 4 illustrates the comparisons of the simulated excess PWP *u* with the analytical solution under different time factors *T*. When the value of *T* is close to zero, it represents the beginning period of the accreted slurry and the filled height is relatively small. With the increase of the time factor *T*, the slurry height will increase at a rate *m* (m/h). Among the continuous filling and consolidating processes, Fig. 4 illustrates only some typical outcomes of PWP at discontinuous certain time factor *T* (= 0.5, 1, 2, 4, 8, 32). The vertical axis represents the ratio of an elevation *z* (m) at any selected position to the current height *h* (m). Along the vertical ordinate value, a value of 0 indicates that the analyzed location is at the base plane of the backfilled stope and a value of 1 indicates that the analyzed location is at the current top surface of the filled slurry. In addition, the horizontal axis represents the ratio of the excess PWP *u* to the effective self-weight stress *γ′* × *h* of the currently filled slurry at different times.

It can be seen from Fig. 4 that there is a good agreement between the numerical and analytical results for the excess PWP across different filling and consolidating periods represented by the dimensionless time factor *T*. The comparisons demonstrate that the constructed numerical model using FLAC3D in this study is a reliable method to investigate the excess PWP and the consolidating process of backfill slurry in mine stopes.

### Simulated stress compared with analytical results of arching model

Together with the validation of the simulated excess PWP* u* for backfill slurry, the effective stress distribution of the consolidating backfill is another key issue to be verified, because the stress near the bottom of the backfill slurry is a crucial factor affecting the barricade stability, and the stresses along the backfill height in stopes are significant for understanding the ground control behaviors of backfill and its strength requirement after side exposure in mining procedures^41–47^.

For fully drained and totally consolidated dry granular materials, the Marston model and the Terzaghi model initially derived from soil mechanics have been widely used to analytically calculate the backfill stress distribution^29,48^. On the basis, after considering the effect of PWP, Li and Aubertin^26^ further proposed a modified analytical solution to assess the total and effective stresses in a submerged cohesionless backfill, which assumed a specified phreatic surface in the backfill to calculate the stresses above and below the phreatic line where the PWP equals to zero.

Equation (6) was suggested by Li and Aubertin^26^ to calculate the effective vertical stress *σ′*_v_ (kPa) at a depth *h* (m) of the backfill in a mine stope with a width *B* (m), a totally filled stope height *H* (m) and a specified phreatic surface at vertical depth *H*_p_ (m) from the top surface of backfill.6

where *γ*_subf_ (= *γ*_satf_ − *γ*_w_, kN/m^3^) is the unit weight of the submerged backfill under the phreatic surface, *γ*_satf_ is unit weight of saturated backfill, and *γ*_w_ is the unit weight of water; *γ*_mf_ (kN/m^3^) is the unit weight of wet backfill above the phreatic surface; *K′*_sf_ (= tan^2^(45° − *ϕ*′_f_/2)) is the effective coefficient of lateral earth pressure for the submerged backfill under the phreatic surface and *K*_sf_ (= tan^2^(45° − *ϕ*_f_/2)) is the active coefficient of lateral earth pressure for the wet backfill above the phreatic surface; *ϕ*′_f_ is effective friction angle of the submerged backfill under the phreatic surface and *ϕ*_f_ is the friction angle of wet backfill above the phreatic surface.

When the backfill is completely under water at the moment of the stope void being fully filled, implying that the assumed phreatic surface is at the top of the backfill (i.e., *H*_p_ = 0), the effective vertical stress *σ′*_v_ (kPa) of the submerged consolidated backfill can be further determined by Eq. (7). This equation can be used as an analytical reference to validate the numerically simulated effective vertical stress of the slurried backfill at different accreting and consolidating times.7

To validate the numerical program of simulating effective stresses in the backfill slurry during consolidation, the same condition corresponding to Eq. (7) was numerically modeled with FLAC3D. According to Fig. 2, the rockmass width *W* was set to zero. The hydraulic boundaries were set to a fixed zero PWP (free drainage) at the top surface of the filled slurry, and fixed impermeable side and base surfaces (Case0 in Table 2). In addition, the displacement of the base of the backfill was completely fixed in all directions, the two side walls of the backfill were fixed in the normal direction and all elements were fixed in the Y-direction (perpendicular to the paper). The backfill is considered as an elastic–plastic material obeying the Mohr Coulomb criterion. The backfill properties used in the analytical equation and numerical model remain consistent with the values in (Table 1).

**Fig. 5:**
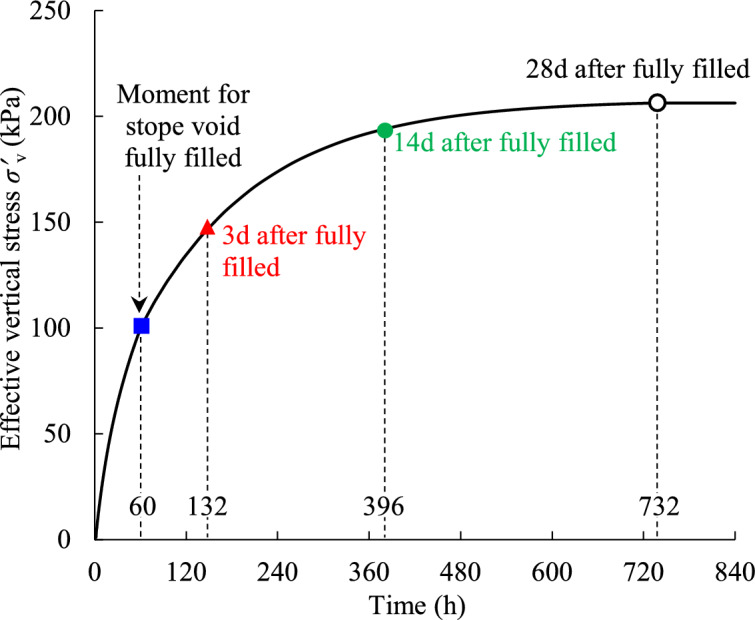
Numerically simulated effective vertical stress monitored at the bottom of the backfilled stope considering the filling and consolidating process.

Figure 5 shows the evolution of the numerically simulated effective vertical stress monitored at the bottom of the stope (illustrated as the blue star monitoring point in Fig. 2a). The curve was started from the beginning of the layer-by-layer accreting increase of the backfill slurry in the excavated stope void, to the moment when the stope void was fully filled over the entire height *H*, and then to the gradual development of the effective stress during the subsequent consolidation of the slurry.

Based on the given program in FLAC3D here (a constant filling rate of 0.5 m/h), it took 60 h to fully fill the stope void to a final height of *H* = 30 m. During this filling period, the effective vertical stress built up as the slurry rises and the PWP was obtained in parallel with the drainage and consolidation process. And it was defined as the 0 day at the moment of the stope void just being fully filled throughout entire stope height *H*, after which the drainage and consolidation will continue to have an effect on the effective vertical stress.

It can be seen from Fig. 5 that the effective vertical stress increases rapidly within the period from 0 to 60 h following the backfill slurry accretion layer by layer in the stope void. After 60 h, the effective vertical stress continues to increase up to 396 h, i.e., 14 days after the stope void is completely filled throughout the entire height *H*. In this period, the increase of the effective stress was mainly caused by the water drainage and gravity conduction from top part slurry to the bottom area of stope. Then, from 396 to 732 h (i.e., 14d to 28d after full filled stope), the effective vertical stress increases marginally, indicating that the water drainage and consolidation process for slurried backfill is coming to an end. And after 28 days starting from the moment of fully filled the stope void, the simulated effective vertical stress at stope bottom hardly changes, which means the backfill in the bottom area of the stope is close to a fully drained and totally consolidated state.

**Fig. 6:**
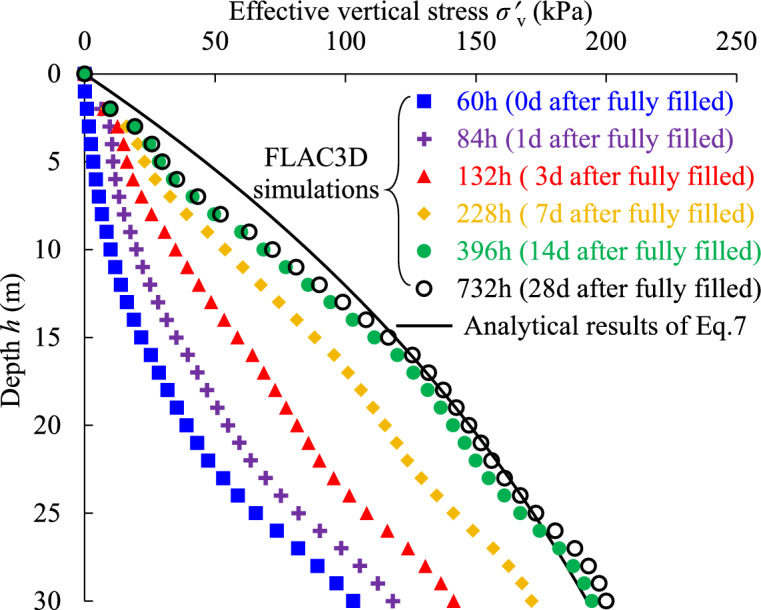
Comparison of analytically calculated effective vertical stress with numerically simulated results at different consolidating times after the stope was fully filled.

Figure 6 illustrates the variations of the effective vertical stress along the vertical center line of the whole height in backfilled stope at different times after the stope was fully filled. In addition, the effective vertical stress calculated with Eq. 7 is also presented as black solid line in Fig. 6 for comparison.

It can be seen from Fig. 6 that the effective vertical stresses simulated with FLAC3D at 60 h are much smaller than the analytical stress with Eq. (7) which is assumed for a submerged fully consolidated backfill. However, it is very interesting to note that with the increase of the consolidating time, the numerically simulated effective stress along the stope height gradually increases and approaches to the analytical solution. These processes clearly illustrate that the gradual self-weight consolidation of the backfill slurry with water drainage from backfill slurry to dissipate excess PWP and increase effective stress. When the time comes to 732 h (i.e., 28d after the stope was fully filled), it can be observed from Fig. 6 that the numerical and analytical results have a good agreement, especially in the lower half height of the backfilled stope. This agreement can also be corresponded to the curve shown in Fig. 5 to illustrate the fully drained and totally consolidated state of backfill near stope floor. In addition, the simulated effective vertical stress in the upper half height of the backfilled stope at the time of 732h is still smaller than the analytical results, which implies that additional time is still needed for the upper slurry to reach the fully drained and totally consolidated state.

The comparisons shown in Fig. 6 demonstrate that the developed numerical program with FLAC3D here is competent to simulate the excess PWP and effective stress states of the slurried backfill in mine stopes at different filling and consolidating periods. Subsequently, the numerical method is applied to investigate the consolidation process of uncemented backfill slurry in a mine stope considering the hydro-geotechnical properties of adjacent rockmass.

## Effect of hydro-geotechnical properties of rockmass on backfill consolidation

### Hydraulic conductivity* k*

**Fig. 7:**
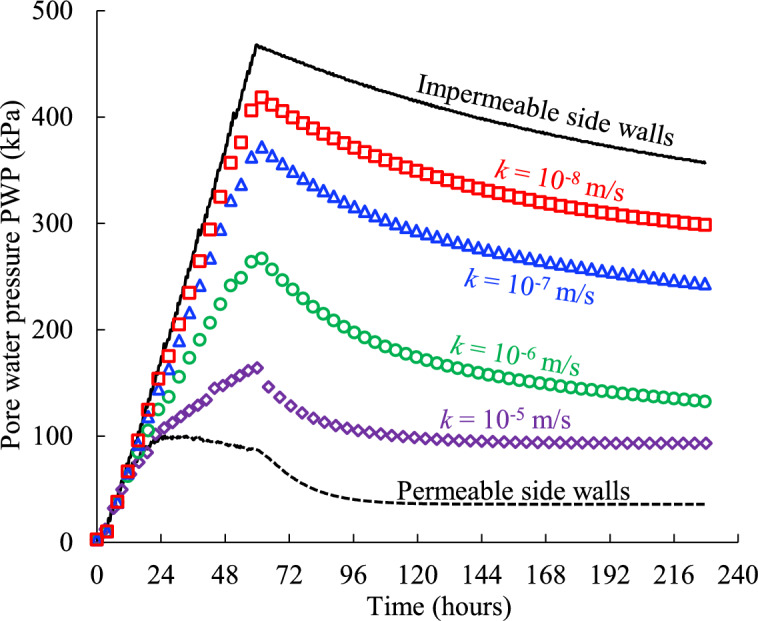
Pore water pressure PWP of the backfill slurry confined by the rockmass with varied hydraulic conductivity* k* (see details in Table 2, Case1).

Figure 7 shows the evolution of the simulated PWP monitored at the bottom (illustrated as the blue star monitoring point in Fig. 2a) of the backfilled stope surrounded by the rockmass with different hydraulic conductivity *k* (Case1 in Table 2). The simulated PWP by numerical models without considering surrounding rockmass but with an idealized impermeable (water-proof) or permeable (free-draining) boundary which were commonly used in previous numerical studies^8,11–15,30,31,49^ is also presented in (Fig. 7) for comparison.

As can be seen from Fig. 7, regardless of the drainage boundary conditions along side walls of the backfilled stope, there are typically four regions of change in the simulated PWP curves as the filling and consolidation time progresses.

1. The first region shows a linear and rapid increase of the PWP from the start point of depositing slurry in the stope void which is mainly caused by the gravity of the poured backfill slurry layer by layer. Within this region, the lower for the permeability along the side drainage boundary, the faster for the PWP increases and the steeper for the obtained curve. For the extreme condition of totally impermeable side walls confining the backfill slurry used in most previous studies^7,8,11–13,15,29^, the steepest slope and longest duration of the linear rise imply the highest rise in PWP monitored at stope bottom.
2. The secondary region carries a marked feature that the continued increasing slope of the PWP curve slows down until to the peak value, accompany with water drainage from the top surface and/or side boundary of filled slurry. It is also worth mentioning that the peak PWP values in the current model under totally impermeable and permeable side drainage conditions are 468 kPa and 102 kPa respectively, and this nearly 5-times difference in peak PWP proves, once again, that it is essential to consider the practical drainage conditions along side walls of backfill slurry, otherwise the simulated PWP of backfill will be over conservative with impermeable side walls or too aggressive with totally free drainage side walls assumed in most previous analytical and numerical models. In addition, Fig. 7 illustrates that the higher for the hydraulic conductivity* k* of rockmass, the smoother for the slope increase to the peak PWP, and this is because the potential to increase pore pressure has been consumed by drainage along the side boundary through rockmass in adjacent stopes.
3. After the peaks, the simulated PWP curves gradually decline to steady states with time, forming the third region. The peak values commonly occur at the moment when the void is fully filled, except the model with totally permeable side walls in which the peak was reached earlier due to the free drainage ability of the side walls. Moreover, it is clear that the smaller the hydraulic conductivity* k* of the rockmass, the slower the PWP decreases until a steady constant PWP is reached, which means the pressure exerted by the backfill slurry at the stope bottom on the barricade structures will last a longer time, which will have an adverse impact on the stability and safety.
4. The fourth region is the final steady state of the simulated PWP which means the poured backfill slurry in stopes has reached a totally drained and consolidated state under specific conditions. From Fig. 7, it can be seen that the ideal model with totally permeable side walls has a constant PWP value about 36 kPa after 4 days (96 h) starting from the original slurry pouring into the stope. Furthermore, it takes about 7 days (168 h) to obtain a steady constant PWP of 94 kPa for the same filled stope but with a hydraulic conductivity* k* = 1 × 10^–5^ m/s of the rockmass. However, the steady constant PWP of the slurry cannot be reached within the simulated 10 days (240 h) for the filled stopes with confining rockmass of hydraulic conductivity 1 × 10^–6^, 1 × 10^–7^ and 1 × 10^–8^ m/s, which means it needs some extra time for the slurry to reach the drained and consolidated state. The smaller the hydraulic conductivity, the longer it takes to go through the consolidation process to a constant PWP in the fourth region.

**Fig. 8:**
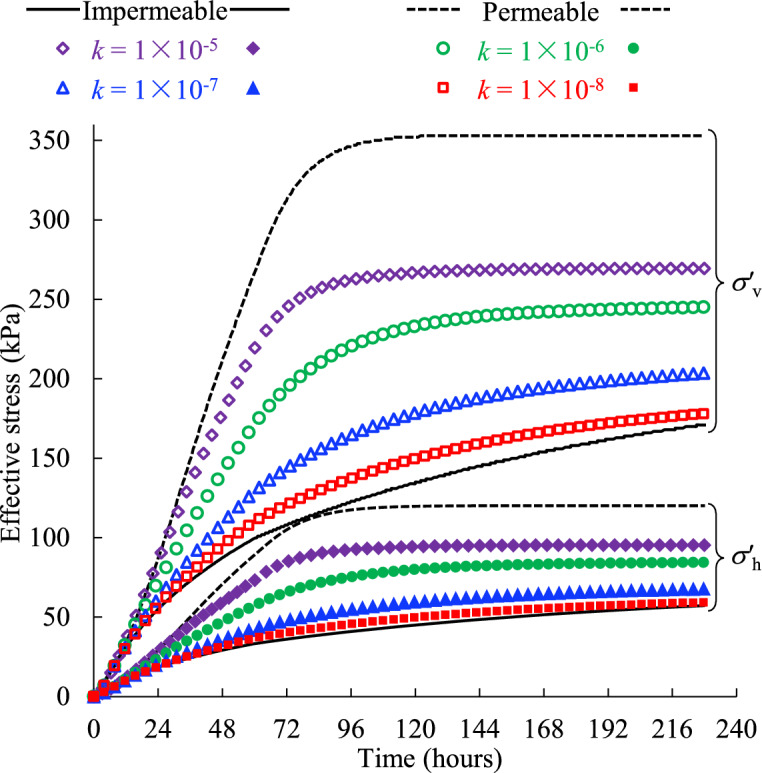
Effective stresses of the backfill monitored near the bottom of the stope confined by the rockmass with varied hydraulic conductivity* k* (see details in Table 2, Case1).

Figure 8 presents the evolution of the effective vertical σ′_v_ and horizontal σ′_h_ stresses of the backfill slurry monitored near the bottom of the stope confined by the rockmass with different hydraulic conductivities. The effective stresses yielded from the idealized models with totally impermeable and permeable side boundaries are also presented for comparison.

It can be seen from Fig. 8 that the vertical and horizontal effective stresses follow the similar upward trend, increasing steadily with time before reaching the plateaus. In addition, with a higher hydraulic conductivity of the side rockmass, both the vertical and horizontal stresses experience a much steeper increment which means the final stable stress state will be reached faster. This, combined with the lower PWP for the higher hydraulic conductivity of the adjacent rockmass shown in (Fig. 7), suggests a load transfer from the pore water to the backfill matrix, which results in the development of effective stresses. Since the rate at which consolidation occurs dictates the rate of stress transfer to the backfill matrix^50^, higher permeability of the surrounding rockmass facilitates the consolidation of the backfill slurry in one stope. If the surrounding rockmass is simplified as the totally impermeable or permeable boundary, unreasonable consolidation rate will inevitably produce as shown in (Fig. 8), which is not conducive to accurately assessing the effective stresses of the backfill slurry in practical stopes.

A smooth increasing trend of effective stresses before reaching a stable value can be seen in (Fig. 8), and there is no distinct peak like the PWP curves shown in (Fig. 7). This is because the increment of effective stresses within the slurry comes mainly from the drainage and consolidation driven by gravity, independent of the filling process. Therefore, once the backfill is placed in the stope, the effective stresses continue to increase smoothly as the drainage occurs. The filling process is equivalent to loading on the top surface of the backfill^50^, and then the increase in load by placing a new backfill layer is carried mainly by the pore water due to its much higher bulk modulus (2 GPa shown in Table 1) compared with that of the backfill (0.48 GPa shown in Table 1), resulting in typically continuous increase in PWP during filling and a peak value at the end of filling. The sensitivity of the PWP to external loading makes it commonly measured in field monitoring during and after stope filling to prevent safety issues, such as liquefaction and barricade failures^22,24^.

In Fig. 8, the model with permeable side wall and a hydraulic conductivity of 10^–5^ m/s in the adjacent rock mass can reach a stabilized value of effective stress, while others cannot. This is similar to the variation of PWP shown in (Fig. 7) and means the backfill has reached a totally drained and consolidated state. In addition, the highest effective stresses of the permeable boundary show more load have been transferred to the backfill matrix, indicating a greater degree of consolidation and more stable backfill structure. Therefore, the permeability of the surrounding rockmass of a backfilled stope should be carefully modeled in numerical simulations, and using an ideally permeable side wall can produce aggressive outcomes.

**Fig. 9:**
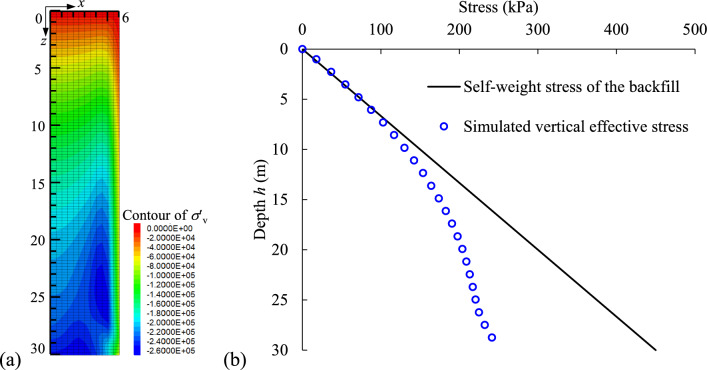
Arching effect developed in the backfilled stope confined by the rockmass with hydraulic conductivity of 10^–6^ m/s at *t* = 228 h: (**a**) contour of vertical effective stress and (**b**) its distribution along the height of stope.

It is interesting to see that the increment of effective stresses in Fig. 8 is smaller than the corresponding changes in PWP in (Fig. 7). For example, the vertical effective stress for *k* = 10^–6^ m/s increases by 5.5 kPa from 144 to 228 h, whereas the PWP decreases by 31.85 kPa during the same period. According to Terzaghi’s effective stress principle, the reduction in PWP should be equal to the increase in effective stress if the total stress remains constant. The discrepancy between the changes in vertical effective stress and PWP can be caused by the arching effect developed in the backfill as shown in (Fig. 9). In Fig. 9a, a “stress arch” forms in the backfill where the vertical effective stress in the middle of the stope (*x* = 0) is greater than that near the right-hand side wall (*x* = 6 m) on the same elevation. Furthermore, the significant difference between the vertical effective stress and self-weight stress of the backfill in Fig. 9b (calculated as *ρ*_df_ × *g* × *h*, where *ρ*_df_ is the backfill density, *g* is the gravitational acceleration, and *h* is the depth of backfill slurry) clearly illustrates the formation of an arching effect developed in the backfill during consolidation. The effect occurs because the settlement of the backfill is restrained by the stiffer adjacent rockmass (the bulk modulus of rockmass and backfill is 2.8 GPa and 0.48 GPa as shown in Table 1). Due to the arching, a portion of the load is transferred to the adjacent rockmass, leading to the observed difference between changes of the effective stress and PWP. The consolidation-induced arching has also been reported by Helinski^12^. In his study, two cases were considered to simulate the consolidation of the backfill slurry during and after filling: a fully rough stope wall (fixed-BC) and a completely smooth stope wall (free-BC). The results show that as consolidation proceeds, some of the vertical stress within the backfill is redistributed to the fixed boundary (arching). This arching prevents further increases in the vertical stress in the fixed-BC case, while the vertical stress in the free-BC case remains similar to the self-weight stress at all stages.

### Porosity ratio *n*

**Fig. 10:**
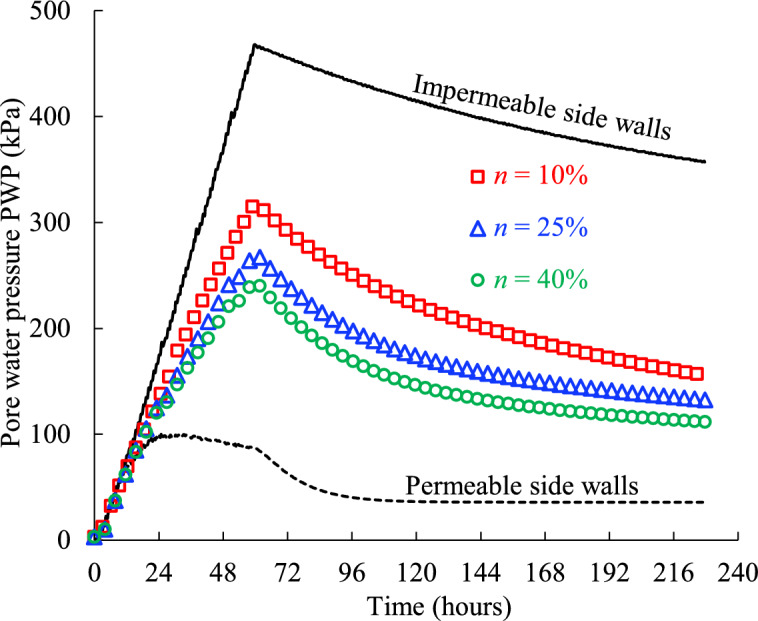
Pore water pressure of the backfill confined by the rockmass with varied porosity ratio *n* (see details in Table 2, Case2).

Figure 10 shows the evolution of the PWP monitored near the bottom of the stope surrounded by the rockmass with porosity of 10, 25 and 40%. The PWP obtained from the models with the permeable and impermeable side walls are also presented.

In Fig. 10, the PWP increases significantly during the filling process, reaching its peak value when the stope is fully filled, after which it continuously decreases. This trend is consistent with the PWP variation in (Fig. 7). It is evident that the PWP decreases as the porosity of the rockmass increases. This can be explained by the fact that a larger porosity of the rockmass provides greater space for accommodating the pore water from the slurry, thereby facilitating the drainage and consolidation of the backfill. Consequently, the PWP decreases as porosity increases.

In practice, the backfilled stopes can be surrounded by either cemented backfill or rockmass. The porosity of cemented backfill typically ranges from 30 to 50%^34,51^, whereas the porosity of rockmass can vary from nearly 0% to as high as 90%, depending on factors such as the mineral composition, depth and geological structures (e.g., joints and fractures)^25^. The voids in the adjacent cemented backfill or rockmass serve as storage space and seepage pathways for the pore water within the backfill slurry during consolidation^52^. In Fig. 10, there is a large gap between the results for backfill confined by the rockmass with varied porosity and those for the impermeable and permeable boundary conditions, underscoring the pronounced impact of the porosity on the PWP. An impermeable boundary means that the adjacent stope has negligible water storage capacity due to very low porosity, while a permeable boundary in numerical simulations assumes unlimited water storage capacity in the adjacent stope. However, neither scenario accurately reflects actual conditions.

**Fig. 11:**
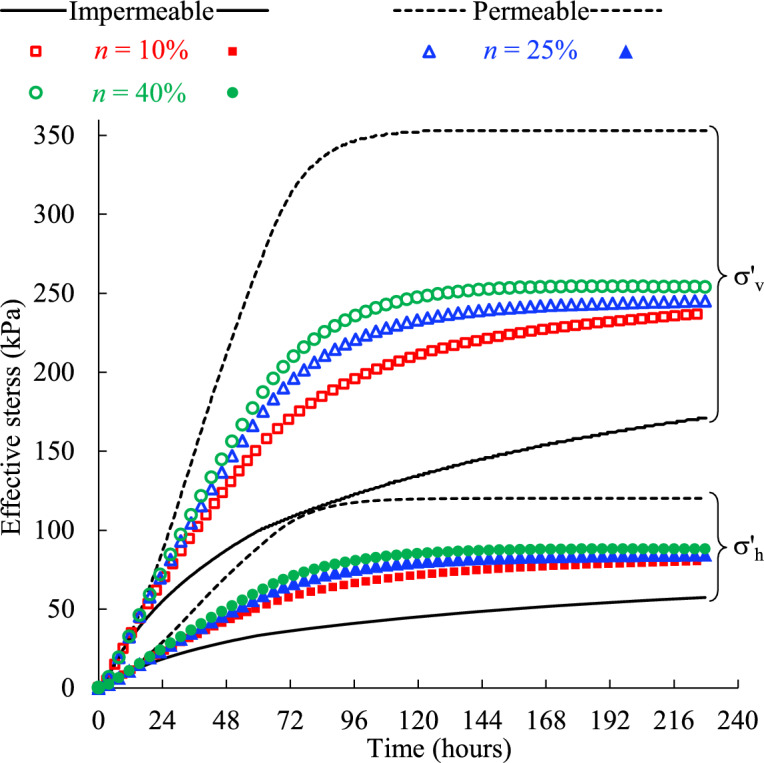
Vertical and horizontal effective stresses of the backfill confined by the rockmass with varied porosity *n* (see details in Table 2, Case2).

Figure 11 presents the evolution of the vertical and horizontal effective stresses of the backfill confined by the rockmass with different porosities. It shows that the effective stresses increase steadily and smoothly once the backfill is placed in stopes. As expected, the effective stresses increase with the porosity of the rockmass. Despite variations in porosity, the effective stresses show similar upward trend but cannot reach an apparent plateau as shown in (Fig. 8). This suggests that the evolution of the effective stresses in (Fig. 11) is not controlled by the porosity of the rockmass. Instead, it is likely dominated by the constant hydraulic conductivity (*k* = 10^–6^ m/s) of the rockmass, which restricts the development of the effective stresses.

### Initial saturation *s*

**Fig. 12:**
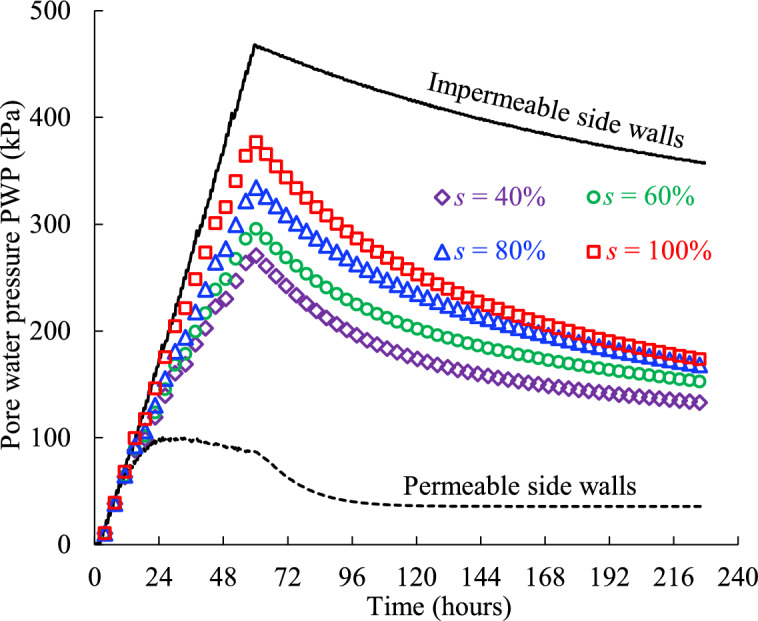
Pore water pressure of the backfill confined by the rockmass with varied initial saturation* s* (see details in Table 2, Case3).

Figure 12 shows the evolution of the PWP monitored near the bottom of the backfill surrounded by the rockmass with varied initial saturation of 40%, 60%, 80% and 100%. Clearly, the PWP increases with the initial saturation of the rockmass. This is because the initial saturation reflects the initial water storage capacity of the rockmass at a given porosity. When the backfill is surrounded by the rockmass with a high initial saturation (e.g., *s* = 100%), the pore water in the backfill cannot flow into the adjacent rockmass promptly, as it takes time for the water originally stored in the surrounding rock to drain out. Conversely, for the rockmass with low initial saturation, which means the greater available water storage capacity, the drainage water of the backfill slurry can more easily enter the rockmass. This leads to enhanced drainage of the backfill and more rapid PWP dissipation during consolidation, resulting in a decrease in PWP as the initial saturation of the rockmass decreases.

Additionally, Fig. 12 reveals that the initial saturation of the rockmass significantly influences the peak value of PWP. For example, the peak PWP increases by 39.2%, rising from 270.53 to 376.74 kPa, as the initial saturation increases from 40 to 100%. This pronounced difference highlights the importance of considering the saturation of the adjacent rockmass when estimating the PWP of the backfill for barricades design. This consideration is particularly critical in extreme mining environments, such as arid desert regions where the rockmass is typically less saturated, or groundwater-rich areas where the rockmass may be fully saturated.

**Fig. 13:**
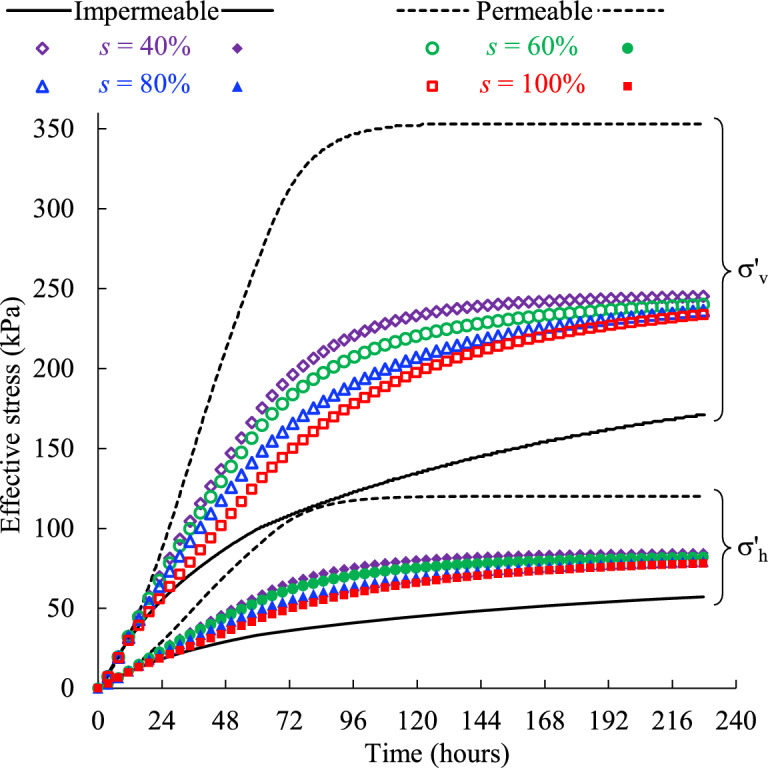
Vertical and horizontal effective stresses of the backfill confined by the rockmass with varied initial saturation* s* (see details in Table 2, Case3).

**Fig. 14:**
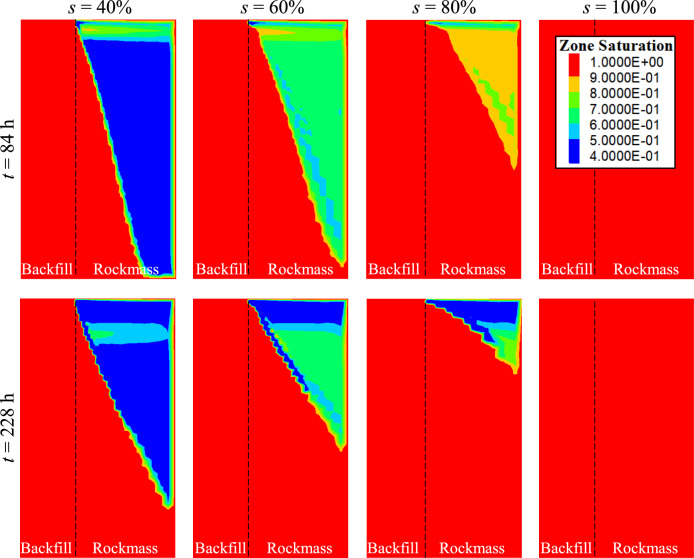
Contours of the saturation at *t* = 84 and 228 h for the rockmass with initial saturation of 40, 60, 80 and 100%.

Figure 13 illustrates the evolution of the vertical and horizontal effective stresses of the backfill slurry confined by the rockmass with varied initial saturations. As expected, the effective stresses increase when the initial saturation decreases from 100 to 40%. In addition, the effective stresses of the backfill confined by rockmass with different initial saturations exhibit noticeable differences between 72 and 144 h after placement, but tend to converge after the 216th hour. This highlights the dynamic influence of the saturation of the rockmass on the development of effective stresses. As shown in (Fig. 14), at *t* = 84 h, a large unsaturated zone exists in the rockmass with the initial saturations of 40 and 60%. This unsaturated zone accommodates the drainage water from the backfill. There is no continuous and fully saturated seepage pathway from the backfill to the right-hand permeable boundary of the rockmass. Consequently, for these two cases, the effective stresses are influenced primarily by the saturation of the rockmass and show an apparent difference. However, for the rockmass with initial saturations of 80% and 100%, the continuous and fully saturated seepage pathways are established. In these cases, the development of effective stresses is mainly controlled by the hydraulic conductivity (*k* = 10^–6^ m/s) of the rockmass, resulting in similar stress patterns. At *t* = 228 h, as more pore water from the backfill infiltrates the rockmass, continuous and fully saturated areas are formed in the rockmass in all cases. At this stage, the effective stresses are controlled by the constant hydraulic conductivity of the rockmass and tend to converge toward a uniform value.

### Rockmass width *W*

**Fig. 15:**
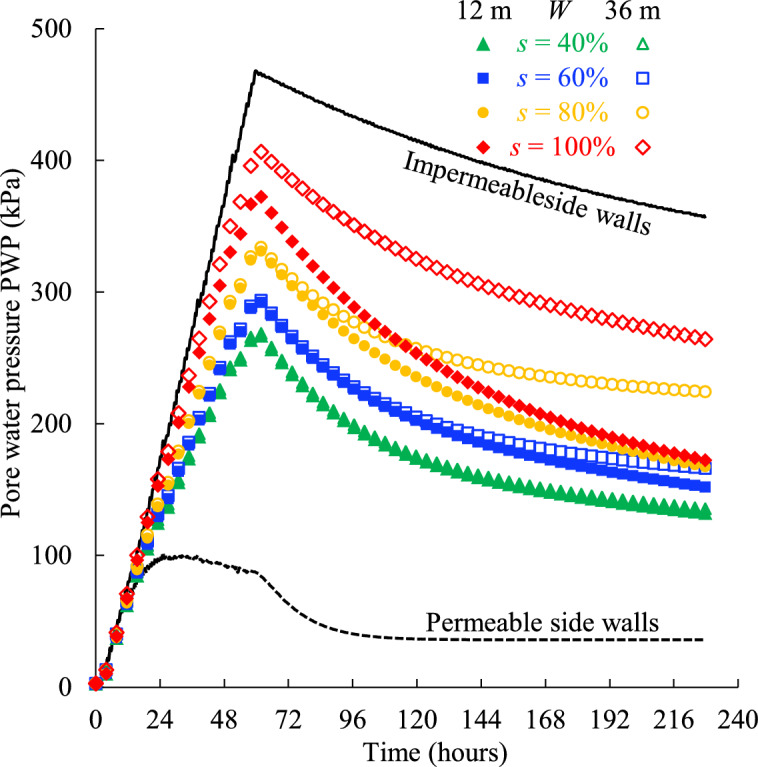
Pore water pressure of the backfill confined by rockmass with varied width* W* and initial saturation *s*.

Figure 15 presents the PWP monitored at the bottom of the backfilled stope confined by the rockmass with different width and varied initial saturation. It is evident that the results for the rockmass width *W* = 12 m and 36 m coincide exactly when the initial saturation of the rockmass is 40% (Table 2, Case 4), indicating the rockmass width has little effect on the PWP evolution under these conditions. To further investigate this phenomenon, additional simulations were conducted in which the initial saturation of the rockmass was varied within the range of 60 to 100%. The resulting PWP values from these simulations are presented in (Fig. 15). Interestingly, differences in PWP between *W* = 12 m and 36 m gradually emerge as the initial saturation of the rockmass increases from 60 to 100%. This demonstrates that the influence of the rockmass width on the PWP evolution of the backfill is closely related to the initial saturation of the rockmass. Specifically, the higher the initial saturation of the adjacent rockmass, the more pronounced the effect of rockmass width on the PWP evolution, as explained below.

**Fig. 16:**
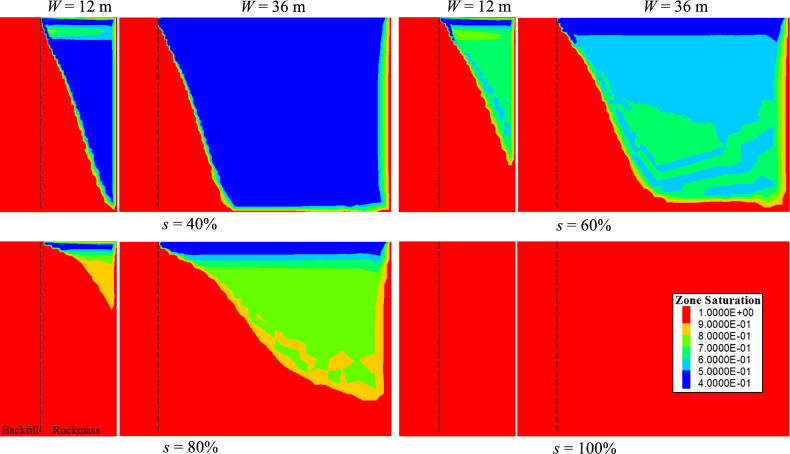
Contours of the saturation at *t* = 132 h for the rockmass with varied initial saturation and width.

Figure 16 shows the saturation contours at *t* = 132 h for rockmass with varying initial saturations and widths. For an initial saturation of *s* = 40%, no continuous and fully saturated seepage pathway forms from the backfill to the right-hand permeable boundary of the rockmass. This is because the unsaturated zones in the less-saturated rockmass (for cases of *W* = 12 m and 36 m) are sufficiently large to accommodate the pore water draining from the backfill, resulting in no discernible difference in PWP between the two cases. For *s* = 60 and 80%, the continuous and fully saturated seepage pathways are established at the bottom of the rockmass. In these cases, the rockmass width begins to influence PWP evolution, as the shorter seepage pathway (*W* = 12 m) facilitates more rapid dissipation of PWP in the backfill compared to the longer pathway (*W* = 36 m). For the rockmass that is initially fully saturated (*s* = 100%), the influence of the seepage length (rockmass width) on the PWP evolution becomes apparent earliest and is most significant.

**Fig. 17:**
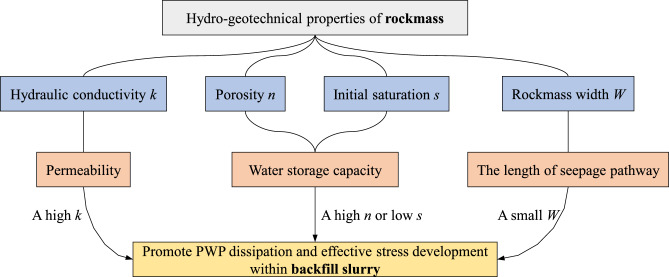
Influence pathways of hydro-geotechnical properties of the surrounding rockmass on PWP and effective stress of backfill slurry.

In summary, Fig. 17 illustrates the influence pathways of hydro-geotechnical properties of the surrounding rockmass on PWP and effective stress of backfill slurry. As discussed above, the hydraulic conductivity of rockmass has the most pronounced effect on the stress state of the backfill slurry during consolidation. While the porosity, initial saturation and rockmass width affect the consolidation process at early stage of stope filling, the hydraulic conductivity governs the PWP dissipation and effective stress development within the backfill slurry in the long term.

## Discussions

### Lateral earth pressure coefficient of consolidated backfill

Lateral earth pressure coefficient *K*′ plays an important role in estimating the stress state within the backfill or the horizontal pressure exerted on barricades. In previous studies, some researchers suggested to use Rankine’s active earth pressure coefficient *K*_a_ = tan^2^(45° − *ϕ*′_f_/2) in analytical solutions^26,44,53^, while others proposed to take Jaky’s at-rest earth pressure coefficient *K*_0_ = 1-sin(*ϕ*′_f_) in some cases^54,55^. To quantitatively understand the *K*′, it is necessary to discuss the effect of hydro-geotechnical properties of adjacent rockmass.

**Fig. 18:**
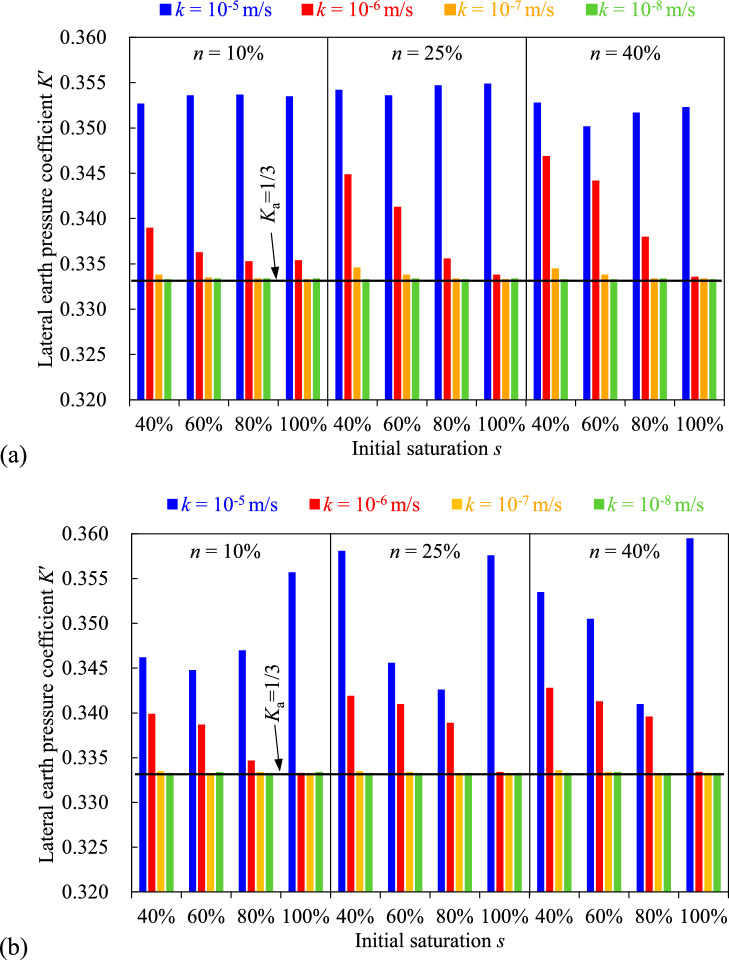
Lateral earth pressure coefficient *K*′ of the backfill confined by the rockmass with varied hydro-geotechnical properties after 228-h consolidating for the case of (**a**) rockmass width *W* = 12 m and (**b**) *W* = 36 m.

The effective horizontal and vertical stresses at the bottom of the backfill after 228 h of consolidation were used to calculate the *K*′ of the consolidated backfill. The results obtained from the backfill surrounded by rockmass with varied hydro-geotechnical properties are also presented in (Fig. 18), with *K*_a_ = 1/3 (calculated using *ϕ*′_f_ = 30°) serving as the benchmark. It was found that the *K*′ of the backfill generally increases with the hydraulic conductivity, regardless of the initial saturation and porosity. In addition, the *K*′ in all cases is closer to the *K*_a_ = 1/3 than to the *K*_0_ = 1/2.

**Fig. 19:**
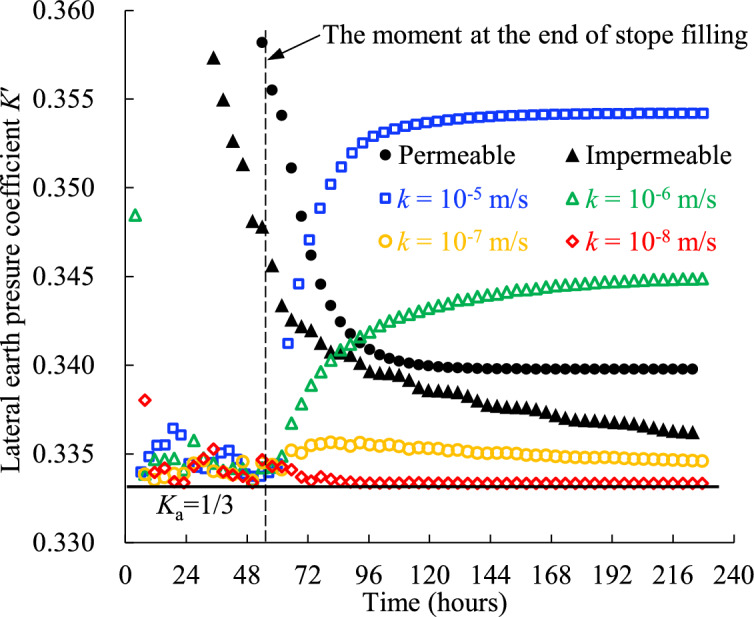
Lateral earth pressure coefficient of the backfill at the bottom of the stope confined by the rockmass with varied hydraulic conductivity *k* (see details in Table 2, Case1).

Figure 19 presents the evolution of *K*′ for the backfill surrounded by the rockmass with varied hydraulic conductivity *k*. The effective vertical and horizontal stresses used to calculate the *K*′ are from the results of Case0 and Case1 in (Table 2).

During the filling process, the *K*′ values for different *k* are similar to each other and close to *K*_a_. After the completion of stope filling, the *K*′ values for *k* = 10^–5^ and 10^–6^ m/s jump sharply and reach stable values, whereas those for *k* = 10^–7^ and 10^–8^ m/s exhibit negligible changes. This shows that the *K*′ of the backfill is not constant but evolves with the filling and consolidation processes. These findings align with the field monitoring results reported by Thompson^22^. In his study, the *K*′ also changes during filling, and the *K*′ at the lower portion of the stope appears to be near *K*_a_ while the upper portion is closer to the *K*_0_. Some numerical simulations conducted without considering the backfill consolidation also show that the *K*′ of the backfill confined by the rockmass depends on its position^54,55^. Therefore, assuming a constant value of *K*′ representative of the entire stope during the consolidation of backfill may be both challenging and potentially unreasonable.

For the results obtained from impermeable and permeable side walls in (Fig. 19), *K*′ shows a markedly different trend compared to cases considering the adjacent rockmass. The *K*′ values for the impermeable and permeable side walls decrease significantly from relatively high values (exceeding 0.5 but not shown in Fig. 19 for clarity) at the start of filling to stable values. This specific trend may be attributed to the mechanical boundary condition of the backfill. When the rockmass is considered, lateral displacement can occur near the right-hand boundary of the backfill because the bulk moduli of the rockmass and backfill are comparable (2.8 and 0.48 GPa, respectively). However, for the backfill with impermeable and permeable side walls, no rockmass is modeled, and the lateral displacement at the right-hand boundary is entirely constrained. The difference in the mechanical boundary condition could lead to different stress state within the backfill, resulting in a distinct evolution pattern for *K*′. Further research is needed to explain this discrepancy.

### Comparison of simulated PWP and stress with in-situ monitored results

To further validate the reliability of the numerical simulation, the simulated results were compared with monitored data for PWP and total stresses. The field monitoring data was obtained from a study conducted at a gold mine, as reported by Helinski^23^. The monitored stope is filled with cemented tailings slurry to a height of 40 m, with plane dimensions of 15 m × 18 m and with a single 6 m wide × 6 m tall draw-point in the middle of the stope length. The filling sequence consisted of filling the first 10 m at a vertical rate of ascent of 0.2 ~ 0.5 m/h, followed by a 24 h rest period. After the rest period, filling continued at a rate of 0.3 ~ 0.6 m/h until the stope was fully filled. A corresponding numerical model similar to that shown in (Fig. 2b) was constructed with FLAC3D. The drawpoint was included in the model, while the adjacent rockmass was excluded and replaced by an impermeable boundary. The backfill slurry was characterized by dry the density *ρ*_d_ = 1220 kg/m^3^, bulk modulus *K*_f_ = 35 MPa, shear modulus *G*_f_ = 18 MPa, internal friction angle *ϕ*_f_ = 35°, cohesion *c*_f_ = 0 and dilation angle *ψ* = 0°, hydraulic conductivity *k* = 5 × 10^–7^ m/s, porosity ratio *n* = 0.5 and initial saturation *s* = 1. These parameters were derived from the laboratory tests conducted in Helinski’s study.

**Fig. 20:**
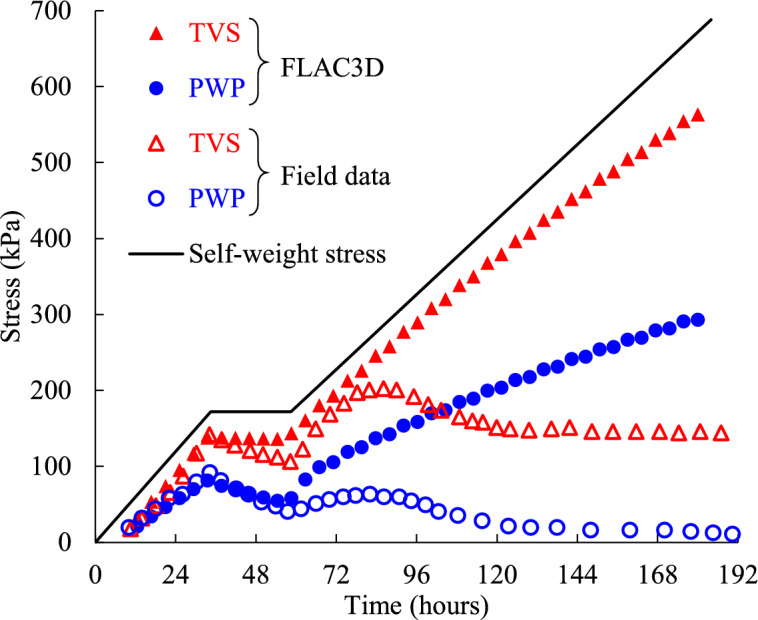
Comparison of simulated results with field monitored values of PWP and total vertical stress.

Figure 20 compares the simulated results with the monitored values of PWP and total vertical stress (TVS), and the self-weight stress of the backfill was also presented. As shown in Fig. 20, from the commencement of the placement of backfill slurry to the end of the rest period, the simulated results are in good agreement with the monitored values. This indicates that the numerical model constructed with FLAC3D is suitable for simulating PWP and TVS at the early age of cemented backfill slurry.

After the rest period, the monitored values of PWP and TVS fluctuated before stabilizing, while the simulation results continued to increase over time, leading to a considerable difference between them. The stabilization of the monitored values of PWP and TVS towards the end of backfilling highlights the effect of cement hydration on consolidation behavior. Some researches have reported that cement hydration can promote PWP dissipation in backfill slurry during consolidation^15,34,56^. However, since the numerical model did not consider the effect of cement hydration, the PWP and TVS continued to increase as the filling proceeds. This discrepancy underscores the limitation of the current numerical model. Nevertheless, the good agreement between the simulated and monitored results during the early age demonstrates the reliability and accuracy of the numerical model.

It should be noted that the numerical model did not consider the influence of the adjacent rockmass, as Helinski’s study lacked detailed descriptions regarding its properties. As discussed earlier, the PWP of the backfill slurry confined by rockmass is always smaller than that obtained from the model with an impermeable side wall. Therefore, in addition to the effect of cement hydration, the gap between simulated results and monitored values in Fig. 20 could also be attributed to the presence of faults, joints or cracks in adjacent rockmass, which were not incorporated into the current numerical simulation.

### Limitations and recommendations for further research

Although the performed numerical simulations are able to evaluate the influences of hydro-geotechnical properties of rockmass in adjacent stopes on consolidation process of backfill slurry, it is necessary to acknowledge the limitations of this work.

The fractured rockmass in the adjacent stope was modeled as an equivalent homogeneous porous medium, which inevitably introduces limitations in capturing the anisotropic seepage characteristics governed by the fractures. While the discrete fracture network (DFN) is commonly employed to represent fracture connectivity and aperture heterogeneity to better simulate fracture-dominated flow paths in rockmass^57^, its computational demands would significantly hinder the efficiency of simulating large-scale backfill consolidation processes. Given that the primary focus of this study lies in the stress and PWP evolutions within the backfill slurry rather than detailed fracture flow path lines in the surrounding rock, the homogenized approximation remains a pragmatic compromise. This simplification gains validity at larger spatial scales, where fracture networks statistically approximate equivalent continuum behavior^58^, aligning with the model’s emphasis on bulk hydraulic characteristics of the rockmass. Nevertheless, future studies targeting fracture-sensitive scenarios should prioritize DFN-integrated modeling to capture the impacts of heterogeneity of the fractured rockmass, thereby enhancing predictive accuracy for critical engineering applications.

In the open stoping with subsequent backfill mining, the primary stope is firstly mined and filled with cemented backfill, while the secondary stope is subsequently excavated and typically filled with uncemented slurry. The numerical simulation here focuses on the consolidation process of the uncemented slurry, excluding the Thermo-Hydro-Mechanical-Chemical (THMC) coupling effects in the cemented slurry. These THMC coupling effects mainly stem from cement hydration, which can significantly affect the consolidation behavior^10^. For example, the formation of hydration products gradually reduces the permeability of the cemented slurry over time, causing it to progressively transition toward a closed system. This reduction in permeability decreases pore water seepage from the slurry into the surrounding rock of adjacent stopes, potentially diminishing the influence of the surrounding rock on the consolidation process of the cemented slurry. However, the impact of multi-physics coupling is not limited to this analysis, and further research focusing on cemented tailings slurry remains necessary for a comprehensive understanding.

In the simulations, the hydraulic conductivity, porosity, and saturation of the rockmass were treated as independent parameters. However, they are inherently coupled in the real rockmass. For instance, the geological faults, joints and other discontinuities in rockmass can lead to increased porosity as well as higher hydraulic conductivity^25^. Experimental results on unsaturated soil also show that hydraulic conductivity varies with changes in saturation and porosity^59^. Clearly, the interdependencies among these parameters can introduce coupling effects on the consolidation process of the backfill slurry. For example, as pore water gradually drains from the slurry into the surrounding rock over time, the hydraulic conductivity of rockmass increases with changes in saturation. These dynamic interactions between parameters result in a time-dependent influence of rockmasses on the consolidation process. Although the current analysis does not capture these coupling and dynamic effects, it still provides a reasonable understanding of the influence of individual parameters of rockmasses on the consolidation behavior of the backfill slurry. Further research is needed to incorporate the interdependencies among these properties to achieve a more comprehensive understanding of the consolidation process of backfill slurry confined by rock masses.

## Conclusions

Numerical simulations were conducted with FLAC3D to investigate evolution of pore water pressure (PWP) and effective stress of uncemented backfill slurry in a vertical stope considering adjacent rockmass with different hydraulic conductivity, initial saturation, porosity, and rockmass width. The main conclusions are summarized as follows.

1. The PWP and effective stresses of backfill slurry in a mine stope considering adjacent rockmass are consistently higher than numerical outcomes obtained by simplifying the rockmass as fully permeable boundaries but lower than those assuming impermeable boundaries. If adjacent rockmass is neglected, the evaluation of backfill slurry consolidation will be either overly conservative with impermeable side walls or excessively aggressive with permeable side walls. To improve the predictive accuracy of the stress state within backfill slurry, it is essential to carefully account for the influence of the adjacent rockmass.
2. A high hydraulic conductivity of adjacent rockmass can facilitate the consolidation process of backfill slurry. When the hydraulic conductivity of adjacent rockmass varies from 10^–8^ m/s to 10^–5^ m/s, there is approximately a five-fold difference in both the peak values of PWP and effective stresses during consolidation. At lower hydraulic conductivity (10^–8^ m/s), the evolution of PWP and effective stresses in backfill slurry during consolidation resembles the behavior observed under the impermeable boundary. Similarly, for cases with high hydraulic conductivity (10^–5^ m/s), the results are close to those for permeable boundary conditions. In such cases, it is reasonable to simplify the surrounding rockmass as a hydraulic boundary.
3. Higher porosity and lower initial saturation of the adjacent rockmass both promote the PWP dissipation and effective stresses development in backfill slurry. However, their influences are less pronounced compared to the effect of hydraulic conductivity. In the early stages of consolidation, noticeable differences in PWP and effective stresses can be observed in backfill slurry confined by the rockmass with varying porosity and saturation. Over time, as the rockmass becomes progressively saturated, these differences diminish, and the values tend to converge where the consolidation of backfill slurry is governed by the hydraulic conductivity. This highlights the necessity of considering of the impacts of adjacent rockmass in arid desert or groundwater-rich regions.
4. The influence of rockmass width on the consolidation process of backfill slurry is associated with the initial saturation of the adjacent rockmass. At a low initial saturation (40%), the rockmass width has minimal effect on the PWP evolution in backfill slurry. However, as the initial saturation of the rockmass increases from 60 to 100%, an increasingly significant impact of the rockmass width on the PWP evolution is observed.
5. The lateral earth pressure coefficient of backfill slurry confined by rockmass is closer to the active earth pressure coefficient than to the at-rest earth pressure coefficient. Moreover, it is not constant but evolves with the filling and consolidation processes.

## Data Availability

The data that support the findings of this study are available from the corresponding author upon request.
